# Usefulness of Smartphone Apps for Improving Nutritional Status of Pancreatic Cancer Patients: Randomized Controlled Trial

**DOI:** 10.2196/21088

**Published:** 2021-08-31

**Authors:** Jiyoung Keum, Moon Jae Chung, Youngin Kim, Hyunyoung Ko, Min Je Sung, Jung Hyun Jo, Jeong Youp Park, Seungmin Bang, Seung Woo Park, Si Young Song, Hee Seung Lee

**Affiliations:** 1 Division of Gastroenterology, Department of Internal Medicine Ewha Womans University College of Medicine Seoul Republic of Korea; 2 Division of Gastroenterology, Department of Internal Medicine Institute of Gastroenterology Yonsei University College of Medicine Seoul Republic of Korea; 3 Department of Biomedical Systems Informatics Yonsei University College of Medicine Seoul Republic of Korea; 4 Noom Korea, Inc Seoul Republic of Korea; 5 Department of Gastroenterology, CHA Bundang Medical Center CHA University Seongnam Republic of Korea

**Keywords:** pancreatic ductal adenocarcinoma, mobile app, nutritional support, quality of life, chemotherapy

## Abstract

**Background:**

Approximately 80% of pancreatic ductal adenocarcinoma (PDAC) patients suffer from anorexia, weight loss, and asthenia. Most PDAC patients receive chemotherapy, which often worsens their nutritional status owing to the adverse effects of chemotherapy. Malnutrition of PDAC patients is known to be associated with poor prognosis; therefore, nutritional management during chemotherapy is a key factor influencing the outcome of the treatment. Mobile apps have the potential to provide readily accessible nutritional support for patients with PDAC.

**Objective:**

We aimed to evaluate the efficacy of a mobile app–based program, Noom, in patients receiving chemotherapy for PDAC.

**Methods:**

We prospectively enrolled 40 patients who were newly diagnosed with unresectable PDAC from a single university-affiliated hospital in South Korea, and randomly assigned them into a Noom user group (n=20) and a non-Noom user group (n=20). The 12-week in-app interventions included meal and physical activity logging as well as nutritional education feedback from dietitians. The non-Noom user group did not receive any nutrition intervention. The primary outcomes were the changes in the nutritional status and quality of life (QoL) from the baseline to 12 weeks. The secondary outcomes included the changes in the skeletal muscle index (SMI) from the baseline to 12 weeks. The European Organization for Research and Treatment of Cancer (EORTC) Quality of Life Core Questionnaire (QLQ-C30) and the Patient-Generated Subjective Global Assessment (PG-SGA) were used as paper questionnaires to assess the QoL and nutritional status of the patients. Intention-to-treat and per-protocol analyses were conducted. Regarding the study data collection time points, we assessed the nutritional status and QoL at the baseline (T0), and at 4 (T1), 8 (T2), and 12 (T3) weeks. Abdominal computed tomography (CT) imaging was conducted at the baseline and after 8 weeks for tumor response and SMI evaluation. The skeletal muscle area (cm^2^) was calculated using routine CT images. The cross-sectional areas (cm^2^) of the L3 skeletal muscles were analyzed.

**Results:**

Between February 2017 and January 2018, 48 patients were assessed for eligibility. Totally 40 patients with pancreatic cancer were included by random allocation. Only 17 participants in the Noom user group and 16 in the non-Noom user group completed all follow-ups. All the study participants showed a significant improvement in the nutritional status according to the PG-SGA score regardless of Noom app usage. Noom users showed statistically significant improvements on the global health status (GHS) and QoL scales compared to non-Noom users, based on the EORTC QLQ (*P*=.004). The SMI decreased in both groups during chemotherapy (Noom users, 49.08±12.27 cm^2^/m^2^ to 46.08±10.55 cm^2^/m^2^; non-Noom users, 50.60±9.05 cm^2^/m^2^ to 42.97±8.12 cm^2^/m^2^). The decrement was higher in the non-Noom user group than in the Noom user group, but it was not statistically significant (-13.96% vs. -3.27%; *P*=.11).

**Conclusions:**

This pilot study demonstrates that a mobile app–based approach is beneficial for nutritional and psychological support for PDAC patients receiving chemotherapy.

**Trial Registration:**

ClinicalTrials.gov NCT04109495; https://clinicaltrials.gov/ct2/show/NCT04109495.

## Introduction

Cancer cachexia is associated with poor therapeutic response, treatment-related adverse events, and low quality of life (QoL) in pancreatic ductal adenocarcinoma (PDAC) patients [1]. Approximately 80% of PDAC patients suffer from a wasting syndrome known as “cancer anorexia-cachexia syndrome,” which is characterized by anorexia, weight loss, asthenia, and poor prognosis [2-7]. Patients with PDAC have high risk of nutritional malabsorption and metabolic problems compared to patients with other types of cancers, as the pancreas plays a crucial role in exocrine and endocrine functions [8-11].

In addition, almost 80% of PDAC patients are not operable at diagnosis and receive palliative chemotherapy [12]. Chemotherapy aggravates anorexia, nausea, vomiting, and abdominal pain, which can also affect the patients’ QoL and nutritional status. Unfortunately, studies on nutritional evaluation and management of PDAC patients during chemotherapy are insufficient. Malnutrition of PDAC patients is associated with chemotherapy-induced toxicity, low adherence to anticancer treatment, as well as poor QoL and survival [2,4,6,13]. Therefore, research on the nutritional management for PDAC patients during active treatment should be considered.

Digital health care systems, especially mobile apps, have the potential to provide readily accessible nutritional and psychological support for cancer patients [14-23]. To date, there has been no randomized controlled clinical trial to evaluate the effectiveness of app-based programs targeting patients with PDAC undergoing chemotherapy. The main purpose of this pilot study was to evaluate the efficacy of mobile app–based supportive care for PDAC patients in the aspects of nutritional status, skeletal muscle index (SMI) change, and QoL.

## Methods

### Study Participants

In this randomized controlled trial (RCT) (Trial number NCT 04109495), the study participants were prospectively recruited at a tertiary hospital in South Korea between February 2017 and January 2018. The inclusion criteria were as follows: (1) males or females aged between 20 and 70 years; (2) patients newly diagnosed with PDAC within the last 3 months and slated to receive chemotherapy, (patients) able to access the Internet through their mobile phones; and (4) patients able to read and write Korean. The exclusion criteria were as follows: (1) history of abdominal surgery within the past year and with plans to undergo abdominal surgery; (2) acute illness or infection status (pneumonia, sepsis, shock, etc.); (3) known chronic liver and obstructive pulmonary diseases; (4) known absorption disorder due to gastrointestinal (GI) mucosal disease (ulcerative colitis, Crohn disease, acute and chronic diarrhea, etc.); (5) severe major illness (heart failure, liver failure, kidney failure on hemodialysis, etc.); (6) pregnancy or breastfeeding; (7) use of steroids within the past month before recruitment; (8) being diagnosed with or suspected of having peritoneal seeding or GI obstruction; and (9) history of consuming nutritional supplements.

### Study Design

**Figure 1:**
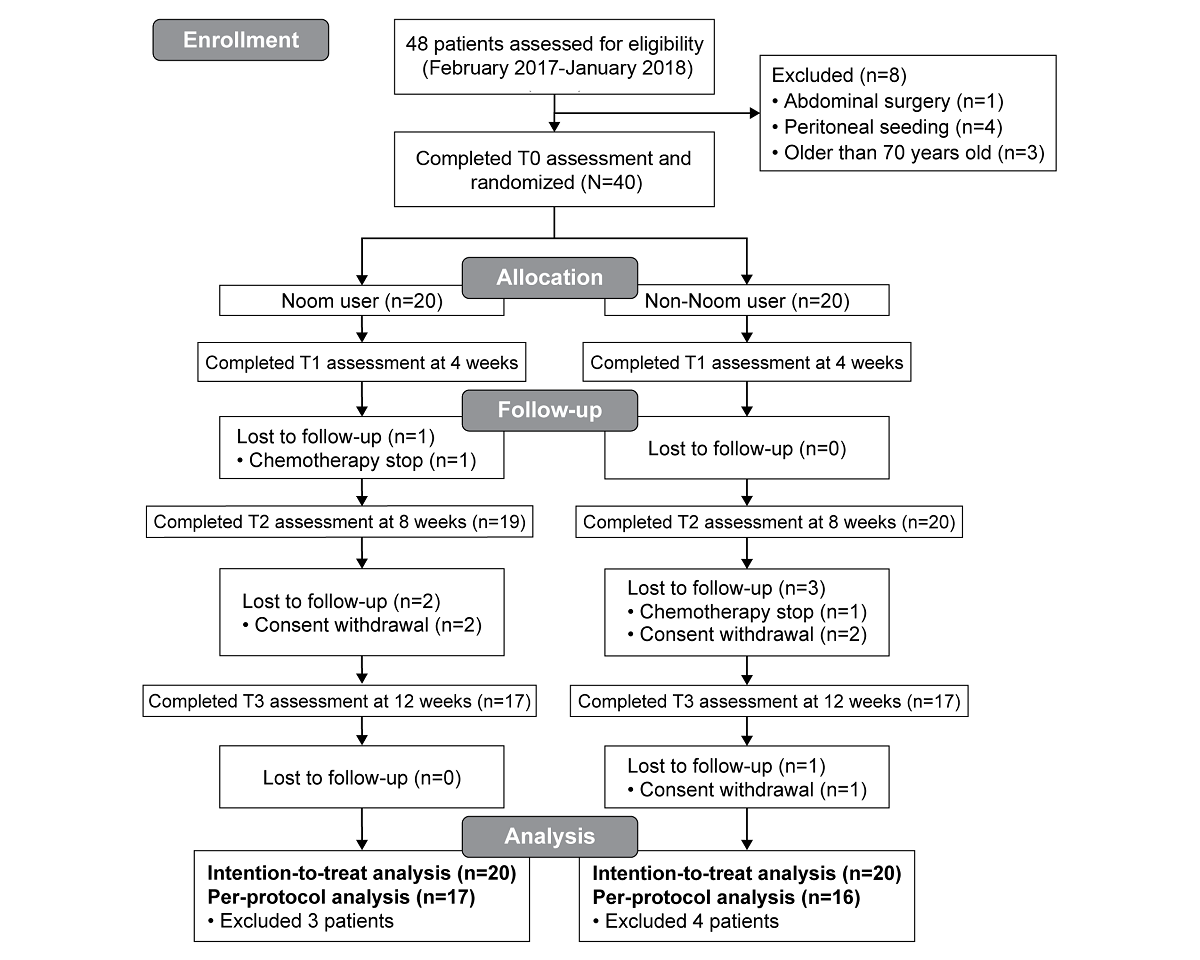
Flow chart of the study recruitment process.

This study was a 12-week prospective, single-center, nonblinded, RCT. The clinicians introduced this study to eligible patients in the clinics, and the researchers met interested patients and confirmed their eligibility. After obtaining written informed consent, all patients were randomly assigned in a 1:1 ratio to the Noom user group or non-Noom user group by the clinical research coordinator (Figure 1). Treatment allocation was performed by the randomized permuted block method using random number tables. As this is a pilot study, we set a target sample size of 40 patients considering the rules of thumb. Browne cites a general flat rule to include at least 30 subjects or more to estimate a parameter [24]. Owing to the nature of the intervention, participant details could not be blinded. This study was approved by the institutional review board of the Severance Hospital (Approval number 1-2016-0061).

### Interventions

Noom (Noom Inc.) is a mobile app for weight management that is commercially available on Google Playstore and Apple Appstore. Noom has a unique curriculum and human coaching intervention, which is widely used in health and fitness apps [25,26]. We adopted this program to the nutritional and behavior intervention for PDAC patients undergoing chemotherapy. The major goal of nutritional intervention was to encourage caloric intake to maintain the nutritional status and QoC of PDAC patients undergoing chemotherapy. Although the commercialized version of the Noom app was designed for weight loss and healthy dietary intake, we used functions such as food logging, step count, weight logging, and messaging for tracking balanced caloric intake and muscle gain. To achieve this goal, the Noom app offered the following interventions: (1) interactive interface with coach–participant messaging, (2) daily articles for basic health knowledge, (3) food logging with color coding, and (4) automated feedback-based food choices (see Multimedia Appendix 1). The articles provided to patients mainly included basic health knowledge, information on how to organize a diet using calorie density, and exercise, and lifestyle information. Patient-specific feedback was provided by the coach. Participants were asked to log their weight by self-report providing information on their meals and physical activity in the app more than 4 days per week.

The coach, who is a clinical dietitian, provided nutritional intervention based on the following goals: (1) Guide participants to consume more calories than the recommended intake calculated by the Harris–Benedict equation [27,28] with additional disease-related energy requirements [29]. (2) Provide more than four feedbacks per week on nutritional intake. (3) Check the participants’ step counts and exercise logs once a week to promote light physical activity. Noom aims to provide nutritional support for PDAC patients through self-management by monitoring their meals and in-app activities. The clinical research coordinator helped the study participants download the Noom app onto their mobile phones and register themselves on the app. A unique username was generated with a personal password. The study participants did not need to pay for accessing Noom.

The participants in both the groups answered paper questionnaires in the presence of the clinical research coordinator and underwent blood tests at the baseline, and at 4, 8, and 12 weeks. The questionnaire items included the gender, age, body weight, type of diagnosed digestive disease, treatment method, status of oral nutritional supplements, and past medical history of the patients. The European Organization for Research and Treatment of Cancer (EORTC) Quality of Life Core Questionnaire (QLQ-C30, version 3.0) and the Patient-Generated Subjective Global Assessment (PG-SGA) were also used to assess the QoL and nutritional status of the patients.

App activity was calculated as the summation of the recorded events, including meals, exercise, weight input, number of messages, and step counts. Participants with app activity for more than 9 weeks were defined to be “above average users” (10/40), and participants with app activity for less than 9 weeks were defined to be “below average users” (7/40). A 9-week period was determined based on the median value of the app users’ activity.

### Comparator

As opposed to the Noom user group, the non-Noom user group did not have access to Noom. This group did not receive any nutrition intervention and attended only study assessments. They received the chemotherapeutic agent as usual. To reduce the bias related to the usually prescribed appetite stimulant, the subjects in both the groups who showed no statistical differences received the same dosage of the appetite stimulant. In this study, patients diagnosed with pancreatic cancer received chemotherapy without regular nutritional intervention.

### Variables

The primary objective was to investigate the changes in the QoL or nutritional status, which were calculated from the EORTC QLQ-C30 and the PG-SGA score, over time according to Noom usage. The EORTC QLQ-C30 is a 30-item cancer-specific questionnaire that incorporates 5 functional scales (physical, role, cognitive, emotional, and social), 3 symptom scales (fatigue, pain, and nausea/vomiting), a global health status (GHS) and QoL scale, several single items assessing additional symptoms commonly reported by cancer patients (dyspnea, loss of appetite, insomnia, constipation, and diarrhea), and the financial impact of the disease [30]. A higher score on the GHS and QoL scales indicates a good QoL, but a higher score on the symptom scales indicates poor QoL.

The PG-SGA is a scoring method for nutritional measurement integrating body weight, food intake, nutritional difficulties and activities, and therapeutic information provided by physicians. A score ≥9 indicates a critical need for nutritional intervention [31]. Several studies have verified that the PG-SGA is a reliable and valid assessment of the nutritional status of cancer patients; therefore, we used the PG-SGA scale as a nutritional status assessment tool [14,32-34]. A trained nurse assessed all the PG-SGA scores to maintain consistency in the test results.

The secondary objective was to observe changes in the SMI according to Noom usage. We evaluated whether the SMI was associated with Noom usage at the baseline and during the follow-up period. The skeletal muscle area (cm^2^) was calculated using routine computed tomography (CT) images through the picture archiving and communication system (PACS), an image system using Image J software (US National Institutes of Health) [35,36]. Cross-sectional areas (cm^2^) of the L3 skeletal muscles were analyzed using Image J. At the L3 level, the field of view included the psoas, paraspinal muscles, and abdominal wall muscles. Currently, the most frequently used landmark in the body composition imaging studies for sarcopenia is the L3 level [37]. We segmented the tissues based on the Hounsfield unit of CT scanning using Image J with assistance from a well-trained and an experienced medical doctor. We had previously published several studies using this method [38,39]. The skeletal muscle area was normalized for height (m^2^) and calculated as the SMI (cm^2^/m^2^) [40].

Age, sex, BMI, Eastern Cooperative Oncology Group performance status (ECOG PS), smoking history, tumor extent, tumor size, chemotherapy regimen, and laboratory characteristics (leukocyte, hemoglobin, platelet, albumin, creatinine, and carbohydrate antigen [CA] 19-9) were also examined.

In this study, we attempted to remove the confounding factors such as steroid ingestion, including appetite stimulants, megestrol, and herbs. Clinicians used to prescribe megestrol to stimulate the appetite of cancer patients. To reduce the confounding factors with respect to appetite stimulants, we prescribed the same dose of megestrol (160 mg/day) for all the enrolled patients, except for those who showed good appetite without stimulants. The prescription was confirmed by the clinical judgment of the attending physician.

### Statistical Analysis

Data are expressed as the median, n (%), or n, as appropriate. Variables were compared using Chi-square tests or Fisher exact tests for categorical data and Student *t* tests for continuous variables to evaluate the statistical significance of the differences in the baseline characteristics between Noom and non-Noom users. Variables related to the in-app actions of the above average and below average user groups were compared using the Mann-Whitney test. The primary outcomes of the nutritional status and QoL, as measured by the PG-SGA and EORTC QLQ, were analyzed using intention-to-treat analysis and linear mixed models. Intention-to-treat analysis with the last observation carried forward was applied to account for missing data. The secondary outcome of SMI was assessed in a per-protocol analysis, using the Mann-Whitney test. *P*<.05 was considered to indicate statistical significance. Statistical analyses were performed using the SPSS (version 23.0, IBM Corp.).

## Results

### Patient Characteristics

Between February 2017 and January 2018, 48 patients were assessed for eligibility. A total of 40 patients were enrolled and randomized into 2 groups (Noom users, n=20; non-Noom users, n=20) (Figure 1). After 7 patients dropped out, 17 Noom users and 16 non-Noom users completed all the follow-ups. Attrition was 18% (7/40 participants), including 2 patients (one in the Noom user group and another in the non-Noom user group) who could not continue chemotherapy owing to severe sepsis or progression of disease, and 5 patients (2 in the Noom user group and 3 in the non-Noom user group) who withdrew their informed consent. The baseline variables in Table 1 did not show a significant difference between participants who were included in the intention-to-treat population (n=40) and per-protocol population (n=33).

The median age was 61.5 years (range 34-78 years), and 25 of the 40 patients (63%) were male. All the recruited patients had unresectable PDAC at the time of diagnosis. The baseline characteristics did not show statistically significant differences between the 2 groups, except for the baseline BMI and hemoglobin. Most of the patients received palliative folinic acid, fluorouracil, irinotecan, and oxaliplatin (FOLFIRINOX) as a first-line chemotherapy (17/20 [85%] of Noom users and 18/20 [90%] of non-Noom users).

**Table 1:** Baseline characteristics of the study population.

Variables^a^		Intention-to-treat analysis (N=40)				Per-protocol analysis (N=33)		
		Noom users (n=20)	Non-Noom users (n=20)	*P* value^b^	Noom users (n=17)		Non-Noom users (n=16)	*P* value^b^
Age, median, years (range)		62 (45-70)	61 (34-78)	.25	62 (45-70)		61.5 (34-78)	.31
Sex, male, n (%)		13 (65)	12 (60)	.99	10 (58.8)		10 (62.5)	.83
Height (m)		1.63±0.09	1.63±0.09	.7	1.63 ± 0.09		1.63 ± 0.08	.95
Weight (kg)		58.4±7.9	63.5 ± 11.3	.18	58.2 ± 8.4		62.8 ± 10.9	.22
BMI (kg/m^2^)		21.91 ± 1.57	23.5 ± 2.72	.03	21.83 ± 1.69		23.46 ± 2.57	.04
**ECOG PS,^c^ n (%)**								
	0-1	17 (85)	18 (90)	.99	14 (82.4)		14 (87.5)	.99
	2-3	3 (15)	2 (10)		3 (17.6)		2 (12.5)	
**Smoking history**								
	Never	16 (80)	14 (70)	.47	14 (82.4)		14 (87.5)	.71
	Former or current	4 (20)	6 (30)		3 (17.6)		2 (12.5)	
DM,^d^ n (%)		5 (25)	6 (30)	.72	5 (29.4)		5 (31.3)	.99
SMI (cm^2^/m^2^)^e^		49.62± 11.62	48.43±9.91	.73	49.08± 12.27		50.60 ± 9.05	.69
WBC,^f^ /μL		6310 (5045-7155)	6,975 (6158-8515)	.13	6310 (4925-7340)		6910 (6158-8045)	.26
Hemoglobin, g/dL		12.3 (10.6-13.6)	13.0 (11.9-14.0)	.04	12.3 (10.6-13.2)		13.0 (12.0- 13.8)	.04
Platelet, 10^3^/μL		204.5 (176.5-353.3)	222 (183-289)	.95	196 (178-335)		237 (196-289)	.85
Albumin, g/dL		3.9 (3.5-4.1)	4.1 (3.5-4.3)	.87	3.9 (3.5-4.1)		3.9 (3.3-4.1)	.74
Creatinine, mg/dL		0.77 (0.53-0.91)	0.67 (0.56-0.82)	.27	0.77 (0.55-0.91)		0.69 (0.52-0.82)	.23
Initial CA^g^ 19-9, U/mL		829.6 (190-2768)	310.1 (40.8-1734.8)	.1	920 (136.6-2716)		310 (27.9-1516.5)	.07
Elevated initial CA 19-9, U/mL		19 (95)	16 (80)	.34	16 (94.1)		12 (75)	.18
**Clinical stage, n (%)**								
	Borderline resectable	6 (30)	6 (30)	.93	4 (23.5)		4 (25)	.61
	Locally advanced	5 (25)	6 (30)		4 (23.5)		6 (37.5)	
	Metastatic	9 (45)	8 (40)		9 (52.9)		6 (37.5)	
Tumor size, cm		4.2±1.3	4.2±2.1	.8	4.2±1.3		4.5±2.2	.65
**Chemotherapy regimen, n (%)**								
	FOLFIRINOX^h^	17 (85)	18 (90)	.99	14 (82.4)		14 (87.5)	.99
	Gem/Nab-paclitaxel^i^	3 (15)	2 (10)		3 (17.6)		2 (12.5)	

^a^Data are presented as n (%) for categorical variables and as median (interquartile range) or mean±SD for continuous variables.

^b^*P* values were calculated by Student *t* tests for continuous data and Chi-square or Fisher exact tests for categorical data.

^c^ECOG PS: Eastern Cooperative Oncology Group performance status.

^d^DM: diabetes mellitus.

^e^The skeletal muscle index was calculated from the muscle cross-sectional area (cm^2^)/height (m)^2^ of the lumbar muscle.

^f^WBC: white blood cells.

^g^CA: carbohydrate antigen.

^h^FOLFIRINOX: folinic acid, fluorouracil, irinotecan, and oxaliplatin.

^i^Gem/Nab-paclitaxel: gemcitabine/nanoparticle albumin–bound paclitaxel.

The baseline characteristics of the Noom participants, above average and below average users, are shown in Table 2. The values in Table 2 were calculated by per-protocol analysis, as patients who dropped out (n=7) did not complete the entire 9-week period, which is the median value of the app users’ activity. The 17 Noom users were divided as follows: 10 above average users and 7 below average users. There were no baseline differences in the sex, age, and baseline BMI between the 2 Noom user groups (Table 2, sex, 59% vs. 60%; age, 62 vs. 62.5 years; baseline BMI, 21.8 vs. 21.5).

**Table 2:** Baseline characteristics of Noom users.

Variables^a^		Noom users (N=17)	Above average users (n=10)	Below average users (n=7)
**Baseline**				
	Sex, male, n (%)	10 (58.8)	6 (60)	4 (57.1)
	Age, median, years (range)	62 (45-70)	62.5 (45-70)	62 (58-65)
	Height (m)	1.63±0.09	1.64±0.1	1.61 ± 0.08
	Weight (kg)	58.2±8.4	58.32±8.61	58.04±8.75
	BMI (kg/m^2^)	21.83±1.69	21.54±1.46	22.24±2.02

^a^*P* values were calculated by the Mann-Whitney test between the two user groups.

In the intention-to-treat population, 15 of the 20 Noom users (75%) and 14 of 20 non-Noom users (70%) received megestrol, but there was no significant difference in the number of prescribed patients (*P*=.72) and the total dose of the drug (9,440 mg vs. 12,240 mg, *P*=.06). In the per-protocol population, 12 of the 17 Noom users (71%) and 13 of the 16 non-Noom users (81%) received metestrol, but there was no significant difference in the number of prescribed patients (*P*=.69) and the total dose of the drug (11,120 mg vs. 12,320 mg, *P*=.09).

### Improvement of Nutritional Status Through Mobile App Usage

**Figure 2:**
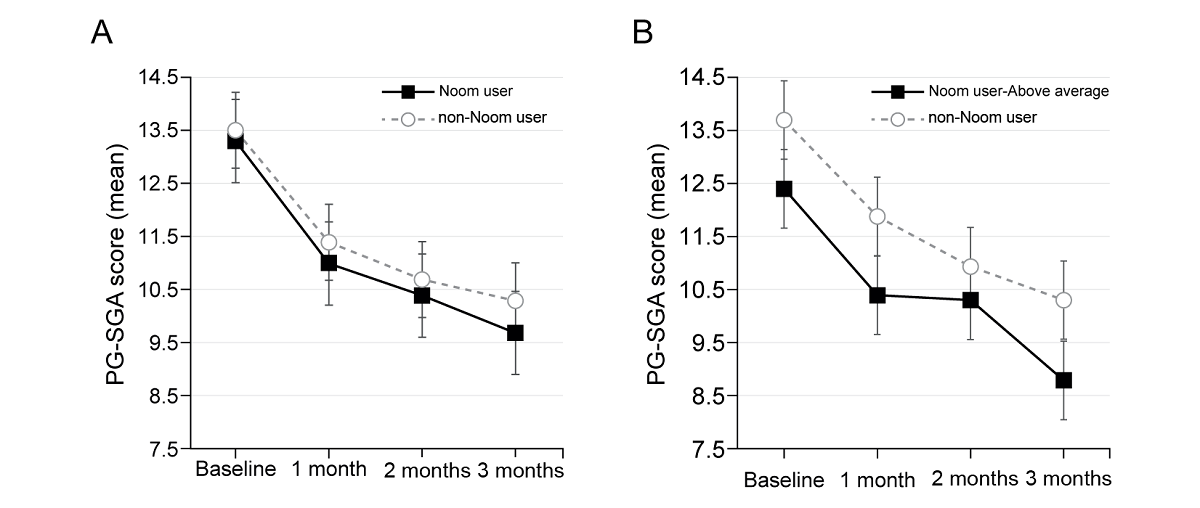
Change in PG-SGA score according to Noom usage over time. (A) All study participants show an improvement in nutritional status according to the PG-SGA score (*P*=.001). (B) All Noom users show improvement in their nutritional status according to the PG-SGA score regardless of their app activity differences. Above average users showed significant improvement in PG-SGA score (*P*=.03). PG-SGA: Patient-Generated Subjective Global Assessment.

In the intention-to-treat analysis, all the study participants showed a significant improvement in the nutritional status according to the PG-SGA score regardless of Noom app usage (Figure 2A, *P*=.001). In the per-protocol analysis, the above average users showed a significant improvement in the PG-SGA score (Figure 2B, *P*=.03).

There were significant differences in the total protein and energy intakes between the above average and below average users (Table 3, 1.3 vs. 1g/kg/day, *P*=.02; 25.2 vs. 17.7 kcal/kg/day, *P*=.04). In the per-protocol analysis, 7 of the 10 above average users (70%) met the individual minimum protein intake requirement, and 6 of the 10 above average users (60%) met the individual minimum energy intake requirement. However, none of the below average users met the minimum recommended daily intake of protein and calories.

The above average users documented meal data more frequently in the Noom app (15.4 meals per week vs. 5.06 meals per week) and showed an increase in their body weight and BMI compared to the below average users (Table 3, body weight, +1.16% vs. -4.43%; BMI, +0.21 vs. -0.81).

**Table 3:** Effects of Noom app intervention on dietary intake, weight, BMI, and engagement characteristics.

Variables		Noom users (N=17)	Above average users (n=10)	Below average users (n=7)
**12 weeks**				
	Total protein intake(g/kg/day)	1 (0.6-1.4)	1.3 (0.9-1.6)^a^	1 (0.5-1)
	Total energy intake(kcal/kg/day)	19.9 (13.9-26.8)	25.2 (17.5-32.7)^b^	17.7 (12.1-20.8)
	Weight loss (kg)	-0.66±4.31	0.68±4.80^c^	-2.57±2.76
	Weight loss (%)	-1.14±7.55	1.16±8.31	-4.43±5.21
	BMI change (kg/m^2^)	-0.21±1.43	0.21±1.60^d^	-0.81±0.93
**In-app actions^e^**				
	Meal input frequency (meals per week)	11.15±7.69	15.41±6.76^f^	5.06±3.95
	Total exercise input frequency (every 12 weeks)	3.35±8.19	5.6±10.28^g^	0.14±0.38
	Articles read (articles/week)	0.98±1.84	1.47±2.3	0.27±0.33
	Number of weight inputs (times/week)	0.6±0.72	0.83±0.7	0.27±0.65
	Messages to coach (messages/week)	5.14±5.56	6.19±4.35	3.63±7.03
	Steps recorded (steps/week)	17,168.23±20,718.02	23,999.58±24,595.55^h^	7,409.17±6,951.79

^a^There was a significant difference in the total protein intake during the 12 weeks between above average and below average users; *P*=.02. The *P* value was calculated by Mann-Whitney tests.

^b^There was a significant difference in the total energy intake during the 12 weeks between above average and below average users; *P*=.04.

^c^There was no significant difference in the changes at 12 weeks between the above average and below average users; *P*=.10.

^d^There was no significant difference in the changes at 12 weeks between the above average and below average users; *P*=.09.

^e^*P* values were calculated by Mann-Whitney tests between the 2 user groups.

^f^All the changes from the baseline to 12 weeks were significant in the above average and below average users; *P*=.007.

^g^All the changes from the baseline to 12 weeks were significant in the above average and below average users; *P*=.01.

^h^All the changes from the baseline to 12 weeks were significant in the above average and below average users; *P*=.02.

### Improvement in QoL Through Mobile App Usage

**Figure 3:**
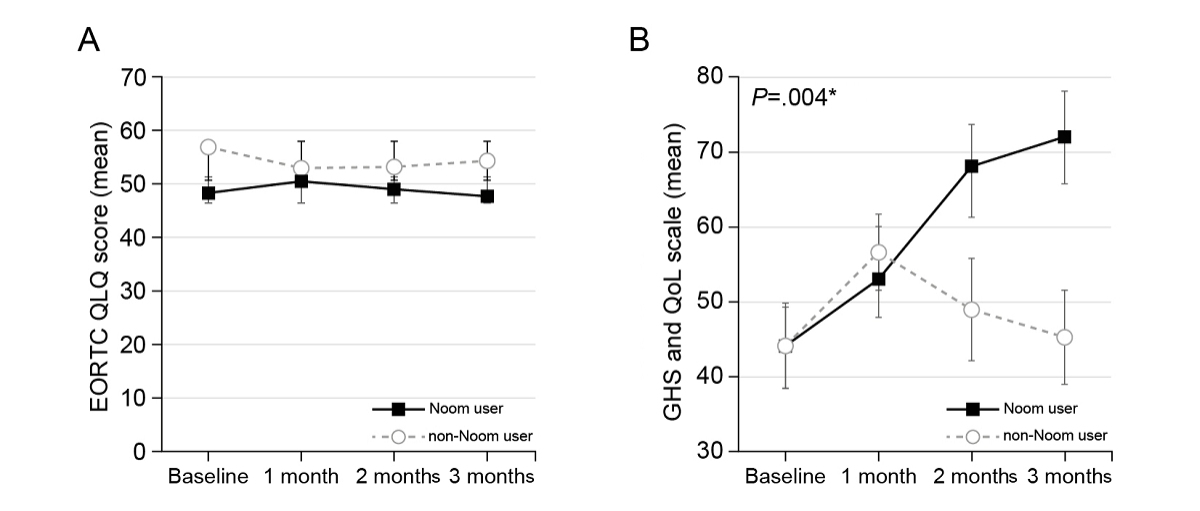
Change in EORTC QLQ-C30 scores according to Noom usage over time. (A) There was no statistically significant difference in EORTC QLQ score between the Noom users and non-Noom users. (B) Noom users showed more statistically significantly improvement on the GHS and QoL scale compared to the non-Noom users over time (*P*=.004). *P values were calculated by the linear mixed model. EORTC QLQ-C30: European Organization for Research and Treatment of Cancer Quality of Life Core Questionnaire; GHS: global health status; QoL: quality of life.

There was no statistically significant difference in the EORTC QLQ score between the Noom users and non-Noom users (Figure 3A). However, on the GHS and QoL scale, there was a statistically significant improvement in the Noom user group compared to the non-Noom user group during the study period (Figure 3B, *P*=.004).

### Skeletal Muscle Change After Mobile App Usage

**Figure 4:**
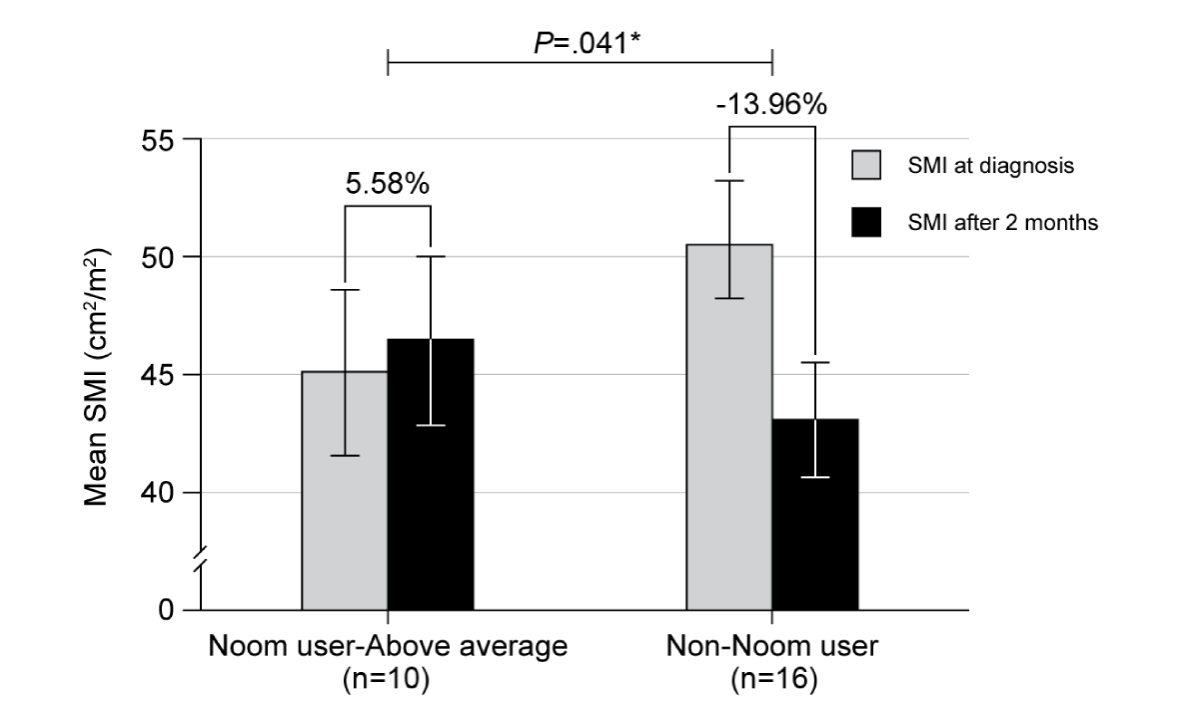
Change in SMI from baseline at 8 weeks. Above average users showed a statistically significant increase in muscle mass after 2 months of Noom usage compared to non-users (*P*=.04). *P values were calculated by Mann-Whitney tests between the 2 groups. SMI: skeletal muscle index.

When receiving chemotherapy, the SMI decreased in both groups (Noom users, 49.08±12.27 cm^2^/m^2^ to 46.08±10.55 cm^2^/m^2^; non-Noom users, 50.60±9.05 cm^2^/m^2^ to 42.97±8.12 cm^2^/m^2^). The decrement was higher in non-Noom user group than in the Noom user group, but it was not statistically significant (-13.96% vs. -3.27%; *P*=.11). In the per-protocol analysis, there was a statistically significant increment in the SMI of the above average user group compared to the non-Noom user group (Figure 4, +5.58% vs. -13.96%; *P*=.04).

### Chemotherapy Response According to Mobile App Usage

Furthermore, the overall best response to chemotherapy according to Noom usage was also analyzed in the per-protocol analysis. The overall best response was defined as the best response recorded from the start of chemotherapy until disease progression, recurrence, or the start of new chemotherapy sessions. A decrease in the tumor size was more prominent in the above average users than in the non-Noom users, although this was not statistically significant, possibly owing to the small study population (-15.6% vs. -6.3%; *P*=.17).

There were no differences in terms of the progression-free survival, overall survival, and duration of chemotherapy between the Noom users and non-Noom users.

## Discussion

### Principal Findings

Mobile apps have been used in the management of chronic diseases such as obesity and hypertension, as well as exercise measurement; they were rarely used directly in hospital care settings. To the best of our knowledge, this is the first study that evaluates the short-term effects of mobile app–based coaching on the change in the nutritional status, SMI, and QoL during PDAC management. The above average Noom users in this pilot study showed statistically significant improvements in their nutritional status. The SMI significantly increased in the above average users compared to the non-Noom users. Moreover, the Noom users showed statistically significant improvements in QoL compared to the non-Noom users based on the GHS and QoL scales of the EORTC QLQ. These findings showed that PDAC patients who receive chemotherapy could be supported by mobile app–based coaching for improving their nutritional and health conditions.

Previous studies have highlighted the importance of supportive care for patients with advanced cancer, including PDAC. Early palliative supportive care led to significant improvements in nutritional statuses, QoL, depression levels, and symptom burden [41-44]. In particular, malnutrition during chemotherapy can induce various adverse effects in humans owing to dysfunction of the intestinal mucosa, decreased immune function, decreased functioning of major organs such as the liver, kidney, and heart, and changes in drug dynamics [10,45-48]. We hypothesized that early nutritional supervision as a supportive treatment could improve the nutritional and psychological conditions of patients with PDAC. In particular, we used a mobile app with human coaching as a nutritional support tool. Even though both groups showed improvements in the PG-SGA score regardless of the frequency of Noom app usage, the above average users showed significant improvements in the PG-SGA scores after using the app. Britton et al reported that the minimum significant difference for the PG-SGA score was 2 points [49]. In this study, the PG-SGA score improved by more than 2 points at 12 weeks in all groups, indicating clinical significance. Therefore, PDAC patients receiving chemotherapy could benefit from using the Noom app, in addition to the usual palliative care.

There have been some attempts at evaluating the efficacy of mobile apps for assisting cancer patients. One randomized study on 114 women with breast cancer who were starting chemotherapy found that e-support program users had better outcomes at 12 weeks for self-efficacy, symptom interference, and QoL compared to users in the control group [15]. Our study also confirmed that QoL improved after using the mobile app. However, the app content used in the two studies differed. Apps used in the Chinese study focused on self-efficacy, social support, and symptom management for patients, whereas the app used in our study focused on nutritional management. Another systematic review also revealed the benefits of app-based programs on the physical activity level, dietary behavior, and health-related QoL in populations diagnosed with solid tumors [50]. Similarly, in our study, the above average users showed better dietary behavior such as inputting their meal data more frequently, with more of them meeting the individual minimum protein and energy intake requirements compared to the below average users. Furthermore, the above average users showed significant improvement in their nutritional status at 12 weeks based on the PG-SGA score. In the present study, the patients had an average PG-SGA score ≥9 points at the baseline, which indicates that almost all participants needed nutritional intervention [2,6,51].

According to previous studies, malnutrition, weight loss, and sarcopenia are risk factors strongly associated with limited tolerance for chemotherapy, short survival times, and poor QoL in PDAC patients. We calculated the SMI using the sarcopenia measurement method. Sarcopenia was measured by CT-based skeletal muscle area assessment, as in many previous studies [52,53]. Loss of skeletal muscle mass is known to be associated with cancer cachexia [54,55]. In the present study, when undergoing chemotherapy, the above average Noom users showed an increment in the SMI, and there was a significant difference between them and the non-Noom users. We assumed that Noom users would be more motivated to improve nutritional intake while monitoring their food intake through the app, and human coaches who are professional nutritionists may have helped Noom users obtain proper nutrition.

A previous retrospective study suggested that early nutritional intervention may affect the overall survival of PDAC patients undergoing chemotherapy [8]. In this study, nutritional intervention included face-to-face dietary consultation with a dietitian. Several studies reported that malnutrition and sarcopenia are factors that are strongly associated with limited tolerance for chemotherapy [3,11]. Despite the increasing evidence demonstrating an association between the nutritional status and clinical outcomes, there is no standard nutritional management tool for PDAC patients undergoing chemotherapy. Although the overall best response to chemotherapy according to Noom usage was not statistically significant, further confirmatory research is needed to achieve better results.

We did not analyze the factors associated with high usage of the app. Although we did not perform statistical analysis, clinical dieticians who coached the patients considered sex as a relevant factor. Female patients or caregivers were considered to use the Noom app more frequently. Further, male patients under 60 years of age were considered to use the Noom app more often. We believe that further studies on mobile app usage according to the age, sex, and education level of patients and their caregivers, as well as the performance status of the patients will benefit future research on nutritional interventions for cancer patients.

### Comparison With Prior Work

Most of the nutritional studies on pancreatic cancer patients undergoing chemotherapy have been performed with face-to-face interventions [56]. Expert dieticians provided essential dietary suggestions and prescribed oral nutritional supplements. These studies have shown positive outcomes, such as improved weight and QoL [8,57,58]. Bauer et al [57] found that cancer patients who received weekly counseling by a dietitian and were advised to consume protein- and energy-dense oral nutritional supplements showed clinically significant improvements in their nutritional status and QoL. Our findings showed a higher improvement in QoL (median change 33.3 vs. 16.7) but lower improvement in nutritional status based on the PG-SGA score (median change 4.5 vs. 9) than those observed in the study by Bauer et al [57], although a direct comparison between the studies was difficult. However, face-to-face studies have limitations in terms of time and space. Mobile health technology is an innovative way to overcome this limitation. Face-to-face interventions can give feedback only on the day of intervention, but with mobile apps, coaches can provide immediate feedback daily based on the patients’ meal records. In addition, face-to-face nutritional education is unlikely to be implemented universally owing to time and space constraints. On the other hand, it has the advantage of providing personalized education for patients through their meal records using mobile apps.

### Limitations

Our study had several limitations. First, this study could not evaluate the long-term effect of the Noom app, as we conducted this pilot study over a period of only 12 weeks. Further research on the long-term effect of mobile apps on PDAC patients is needed. Second, the study participants were recruited from a single center, and the sample size was small. Furthermore, the requirement of access to the mobile app may have resulted in the participation of a more educated population, potentially limiting the generalization of this study. However, as the number of people familiar with using mobile apps increases over time, it is expected that supportive care using mobile apps could become a promising intervention method. Therefore, additional multicenter-mediated validation is needed to confirm the results of this study. However, one of the strengths of this study was it is the first such study to investigate the use of a mobile app in providing supportive care to PDAC patients. Third, the baseline BMI differed between the Noom users and non-Noom users. The Noom users had a lower BMI at the time of diagnosis compared to that of the non-Noom users. However, the BMI changes in the between the 2 groups did not differ significantly at 12 weeks (*P*=.99). Therefore, the baseline BMI differences between the 2 groups did not affect the results. Furthermore, SMI reduction was more prominent in the non-Noom users than in the Noom users, despite the higher baseline SMI in the non-Noom users. Fourth, we could not measure the nutritional intake of the non-Noom users owing to the study design; therefore, it was not possible to analyze whether the Noom users consumed more calories and specific nutrients compared to the non-Noom users.

### Conclusions

This pilot study demonstrated that a mobile app–based approach for providing nutritional and psychological support could be beneficial for patients with PDAC undergoing chemotherapy. Mobile apps could be useful tools for providing prompt and appropriate nutritional support and monitoring of PDAC patients.

## Appendix

Multimedia Appendix 1 Screenshots of the Noom app.

Multimedia Appendix 2 CONSORT-eHEALTH checklist (V 1.6.1).

## Abbreviations

CA: carbohydrate antigen
CT: computed tomography
ECOG PS: Eastern Cooperative Oncology Group performance status
EORTC: European Organization for Research and Treatment of Cancer
FOLFIRINOX: folinic acid, fluorouracil, irinotecan, and oxaliplatin
GHS: global health status
GI: gastrointestinal
PACS: picture archiving and communication system
PDAC: pancreatic ductal adenocarcinoma
PG-SGA: Patient-Generated Subjective Global Assessment
QLQ-C30: Quality of Life Core Questionnaire
QoL: quality of life
RCT: randomized control trial
SMI: skeletal muscle index
