# Supplementary figures and images for: Usefulness of Smartphone Apps for Improving Nutritional Status of Pancreatic Cancer Patients: Randomized Controlled Trial
Source: JMIR Mhealth Uhealth. 2021 Aug 31;9(8):e21088. doi: 10.2196/21088 (PMC8441607; doi:10.2196/21088)

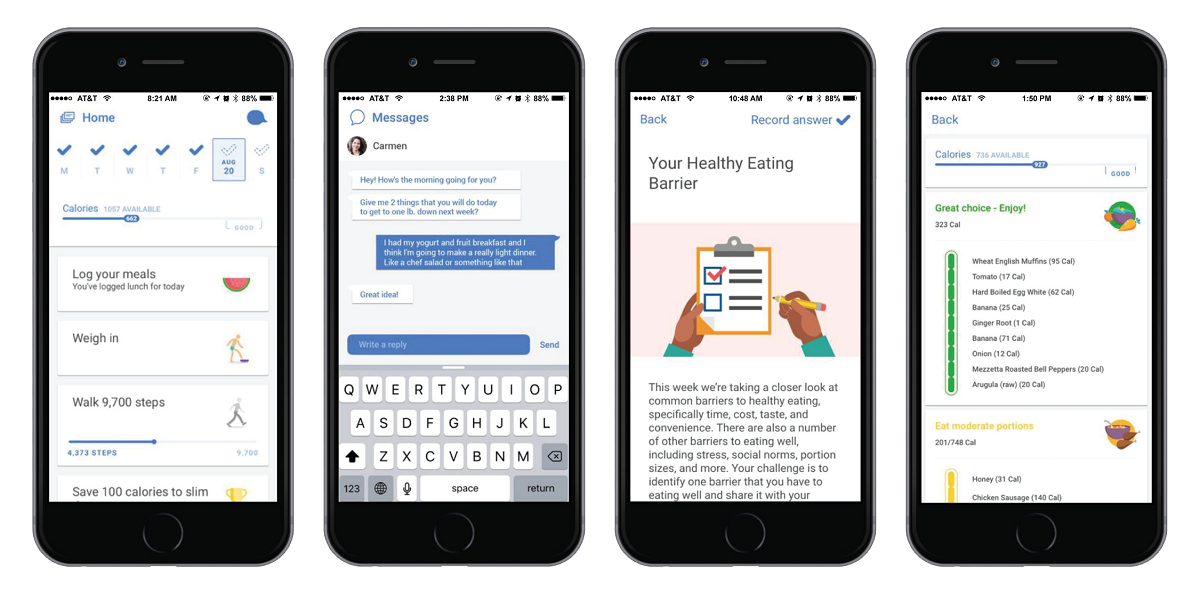

Supplement: Multimedia Appendix 1 [file mhealth_v9i8e21088_app1.png]
